# Clinical features of *Streptococcus intermedius* infection in children: a case series study

**DOI:** 10.3389/fmicb.2023.1207490

**Published:** 2023-08-07

**Authors:** Zhufei Xu, Lichao Gao, Dan Xu, Dehua Yang, Zhimin Chen, Yingshuo Wang

**Affiliations:** ^1^Department of Pulmonology, Children’s Hospital, Zhejiang University School of Medicine, National Clinical Research Center for Child Health, Hangzhou, China; ^2^Department of Cardiology, Children’s Hospital, Zhejiang University School of Medicine, National Clinical Research Center for Child Health, Hangzhou, China

**Keywords:** *Streptococcus intermedius*, child, high-throughput nucleotide sequencing, abscess, lung

## Abstract

**Introduction:**

*Streptococcus intermedius* is an opportunistic pathogen associated with prolonged hospital stays and high mortality rates in adults. However, little is currently known about the clinical features of *Streptococcus intermedius* infection in children.

**Methods:**

This retrospective case series study included 40 children diagnosed with *Streptococcus intermedius*, confirmed through bacterial cultures or high-throughput sequencing. Antibiotic resistance was assessed through susceptibility testing. The site and clinical manifestations were evaluated for all patients.

**Results:**

The common infection sites were the abdominal cavity, skin and soft tissue, intracranial, and invasive pulmonary, with the abdominal cavity being the most frequently affected. The drug susceptibility test showed 100% sensitivity to ceftriaxone, levofloxacin, chloramphenicol, vancomycin, and linezolid, 92.6% sensitivity to penicillin, 73.3% resistance to erythromycin, and 76.7% resistance to clindamycin. Besides antibiotic therapy, surgical intervention or pus drainage was often necessary. Lung imaging of four patients revealed pulmonary abscesses, nodules, or encapsulated pleura. Two cases yielded positive culture results, while three were identified as positive through high-throughput nucleotide sequencing of pleural effusion.

**Discussion:**

In children with *Streptococcus intermedius* infection, emphasis should be placed on the risk of pus or abscess formation. In cases of pulmonary abscess and pleural effusion, especially in male children, *Streptococcus intermedius* should be suspected even if the culture is negative. Improvements in high-throughput nucleotide sequencing are required to reduce misdiagnosis rates.

## Introduction

The *Streptococcus anginosus group* (SAG) is composed of three distinct species: *Streptococcus anginosus* (*S. anginosus*), *Streptococcus intermedius* (*S. intermedius*), and *Streptococcus constellatus* (*S. constellatus*). These species typically inhabit various mucosal sites, including the oral, oropharyngeal, respiratory, gastrointestinal, and genitourinary tracts, and have been established as the most common pathogens responsible for brain abscesses, liver abscesses, and pleural empyema. Despite their medical relevance, there is a significant lack of literature discussing the members of SAG. The growing number of reports on life-threatening infections caused by SAG suggests its emerging pathogenicity. Recent clinical data has highlighted the existence of such infections, characterized by the formation of various types of empyema and abscesses (Pilarczyk-Zurek et al., 2022).

A recent study by Jiang et al. (2020) analyzed 463 specimens that tested positive for *S. anginosus* (*n* = 254, 54.86%), *S. constellatus* (*n* = 173, 37.37%), and *S. intermedius* (*n* = 36, 7.77%). We collected 571 cases of SAG at our hospital between January 2018 and June 2021, including cases of *S. constellatus* (*n* = 388, 67.95%), *S. anginosus* (*n* = 143, 25.04%), and *S. intermedius* (*n* = 40, 7.01%) infection. Therefore, it is estimated that *S. intermedius* accounts for approximately 7–8% of SAG. Among the SAG species, *S. intermedius* is the most pathogenic, often associated with extended hospital stays (Issa et al., 2019) and high mortality rates (Junckerstorff et al., 2014). It is also more likely to lead to abscess formation. Consequently, it is widely thought that *S. intermedius* is more relevant to life-threatening SAG-related infections, although further research is warranted. Pilarczyk-Zurek et al. (2022) summarized the infection rates of the SAG species in previous reports of abscess-forming diseases; *S. intermedius* infections accounted for approximately 50.00% (21/42) of brain abscesses, 10.77% (7/65) of tonsil abscesses, 9.31% (19/204) of oro-facial abscesses, 23.61% (17/72) of lung abscesses, 53.33% (16/30) of empyema cases, and 68.75% (11/16) of liver abscesses. Unfortunately, despite the numerous published cases of *S. intermedius* infection, it remains under-studied, particularly in children compared to other populations, due to low rates of clinical isolation in the past and lack of attention to its pathogenic status.

In this study, we analyzed children infected with *S. intermedius* treated at our hospital over the past 3.5 years. We also conducted a detailed analysis of their clinical characteristics.

## Materials and methods

### Patients

This retrospective case series study included 40 children with *S. intermedius* treated at Children’s Hospital, Zhejiang University School of Medicine, between January 2018 and June 2021. The inclusion criteria were confirmation of *S. intermedius* based on bacterial cultures or high-throughput sequencing and availability of complete clinical and imaging data. The exclusion criteria included contaminated and colonized strains, cases with incomplete data, and outpatient cases.

This study received approval from the Medical Ethics Committee of Children’s Hospital affiliated with Zhejiang University School of Medicine (2022-IRB-087).

### Diagnostic criteria for *Streptococcus intermedius*

The diagnostic criteria for *S. intermedius* were as follows: (1) positive cultures for *S. intermedius* from sterile site fluids, abscess extraction fluid which were obtained during surgery or abscess puncture and drainage after thorough disinfection; (2) positive blood cultures for *S. intermedius* from two or more different sites simultaneously; (3) positive cultures for *S. intermedius* from bronchoalveolar lavage solution, ophthalmic secretions, submandibular gland secretions, no other pathogens were found in the culture, and the clinical manifestations and treatment were consistent *S. intermedius* infection. (4) Positive high-throughput sequencing for *S. intermedius* in sterile site body fluids and no other pathogens were detected, the clinical symptoms were consistent with *S. intermedius* infection and responded well to treatment.

### *Streptococcus intermedius* isolation, culture, identification, and susceptibility test

Isolation and culture of *S. intermedius* were performed as follows: pleural effusion, blood, cerebrospinal fluid, and abdominal effusion were inoculated into culture bottles, while bronchoalveolar lavage fluid and sputum were inoculated on Columbia blood agar plates. Both were incubated in a 35°C incubator with 5% CO2.

Identification and susceptibility testing of *S. intermedius* strains were conducted as follows: suspicious colonies on Columbia blood agar plates were selected, and bacterial identification was performed using Matrix-assisted laser desorption/ionization-time of flight mass spectrometer mass spectrometry (MALDI-TOF, Bruker, Germany). After further isolation and purification, susceptibility testing was carried out using the disc diffusion method, and the results were interpreted according to the latest version of the American Committee of Clinical and Laboratory Standards guidelines. *Streptococcus pneumoniae* (ATCC49619) was used as the quality control strain.

After identifying cases with positive bacterial culture or high-throughput sequencing for *S. intermedius*, their clinical characteristics were retrospectively analyzed. Follow-up data were evaluated through electronic medical records and telephone interviews prior to writing this article. The social skills of the patients with intracranial infection were assessed with the “Infant-Junior Middle School Student’s Ability of Social Life Scale” through telephone interviews. The standard scores ranges is from 6 to 12. The standard scores below 8 is deficiencies, 9 is borderline deficiencies, 10 is normal ability and above 11 is excellent (Wang et al., 2019).

### Assessment of clinical features for *Streptococcus intermedius*

The clinical evaluation, including vital signs, fever, clinical signs and symptoms indicative of infection, laboratory examination (routine hematological and biochemical tests, as well as inflammatory biomarkers such as C-reactive protein (CRP) and procalcitonin (PCT)), and imaging by computed tomography (CT) or ultrasound scanning, were performed based on clinical indications according to local protocols.

### Statistical analysis

Measurement data that followed a normal distribution were presented as “mean ± standard deviation (SD)” and independent sample t-tests were used for hypothesis testing. Measurement data that did not follow a normal distribution were expressed as median (interquartile range), and non-parametric tests (Mann–Whitney U test, Kruskal-Wallis H test) were used for hypothesis testing. Counting data were presented as the number of cases and percentage (%). Statistical analysis was performed using SPSS 16.0 software. A two-sided, and value of *p* <0.05 was statistically significant.

## Results

### Study population

**Figure 1:**
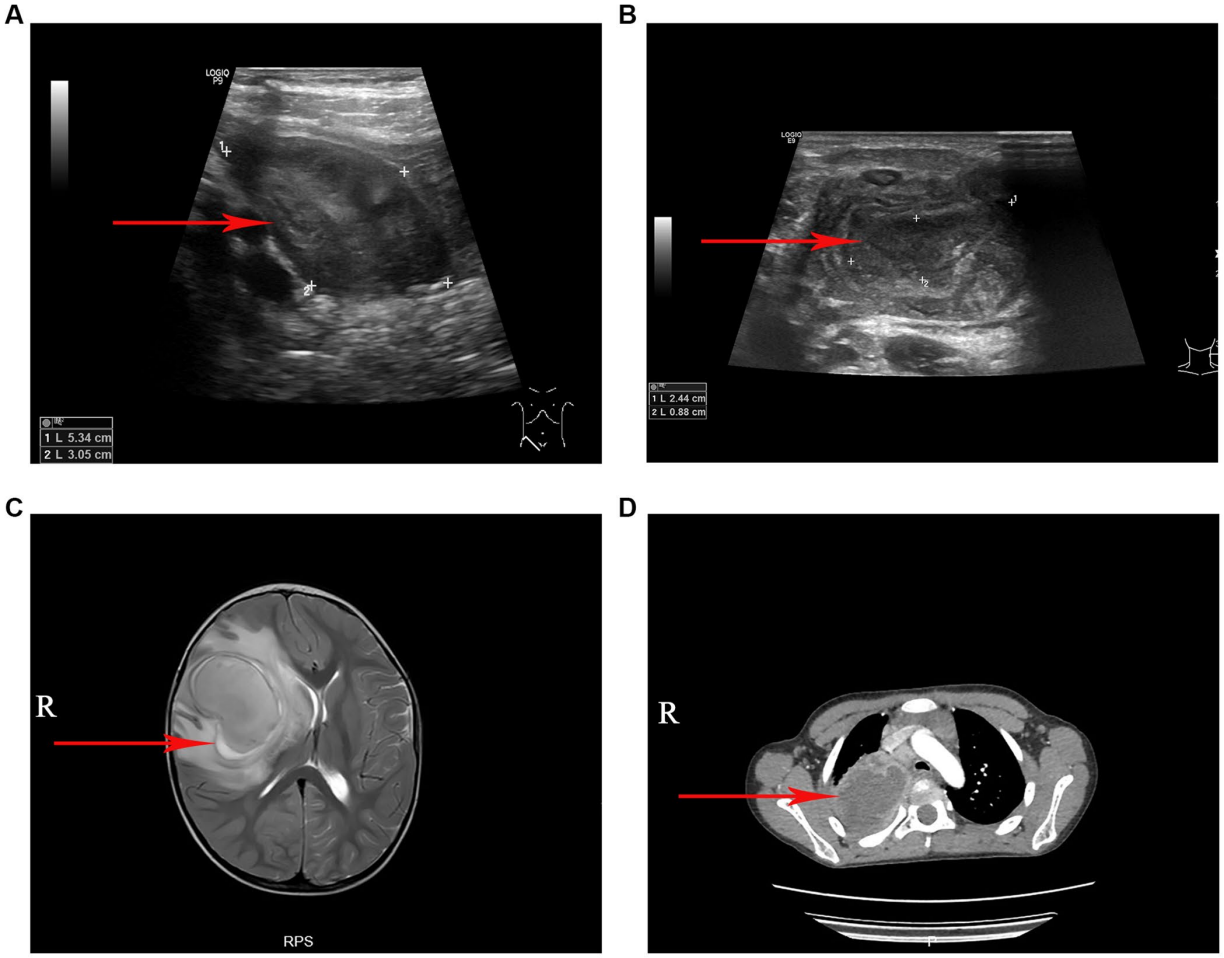
The imaging manifestations of *S. intermedius*. **(A)** Ultrasound examination revealed a local abscess formation around the appendix. **(B)** Ultrasonography revealed a cervical lymph node abscess formation. **(C)** Magnetic resonance imaging (T2-weighted) showed a large cerebral abscess in the right cerebral hemisphere. **(D)** Chest computed tomography showed a pulmonary abscess in the upper lobe of the right lung with surrounding inflammatory changes.

A total of 40 patients who tested positive for *S. intermedius* through bacterial culture or high-throughput sequencing were screened. The general information of these children were showed in Table 1. Radiographic or ultrasonic evaluations showed predominately local abscess or mass formation (Figure 1). Bacterial cultures were obtained from various specimen types, including 36 cases of pus, 2 cases of pleural fluid, 1 case of bronchoalveolar lavage fluid, and 1 case of cerebrospinal fluid. High-throughput nucleotide sequencing was performed on 3 cases of pleural fluid (sequence numbers 3, 110, and 1,289), 1 case of bronchoalveolar lavage fluid (sequence number 302), and 1 case of cerebrospinal fluid (sequence number 75). *S. intermedius* was found to be co-infected with other bacterial infections in 17 cases (42.50%), with *Escherichia coli* being the most common co-infecting pathogen (14 cases, 35.00%).

**Table 1:** General information on children with *S. intermedius* infection.

Item	Number
Number of children	40
Number of boys (% patients)	23 (57.5%)
Average age in month (min, max)	60.8 (0.9, 164)
Average length of hospital stay in days (min, max)	8 (1,60)
Fever	22 (55%)
Number of fever course (min, max)	3 (1, 14)
Specimens of bacterial culture	
Pus	36
Pleural fluid	2
Bronchoalveolar lavage fluid	1
Cerebrospinal fluid	1
Specimens of high-throughput nucleotide sequencing	
Pleural fluid	3
Bronchoalveolar lavage fluid	1
Cerebrospinal fluid	1
Number of co-infections (% patients)	
*Escherichia coli*	14 (35.00%)
*Pseudomonas aeruginosa*	2 (5.00%)
*S. constellatus*	1 (2.50%)
*Staphylococcus aureus*	1 (2.50%)
*Proteus vulgaris*	1 (2.50%)
*Enterococcus faecium*	1 (2.50%)

### Susceptibility test

**Figure 2:**
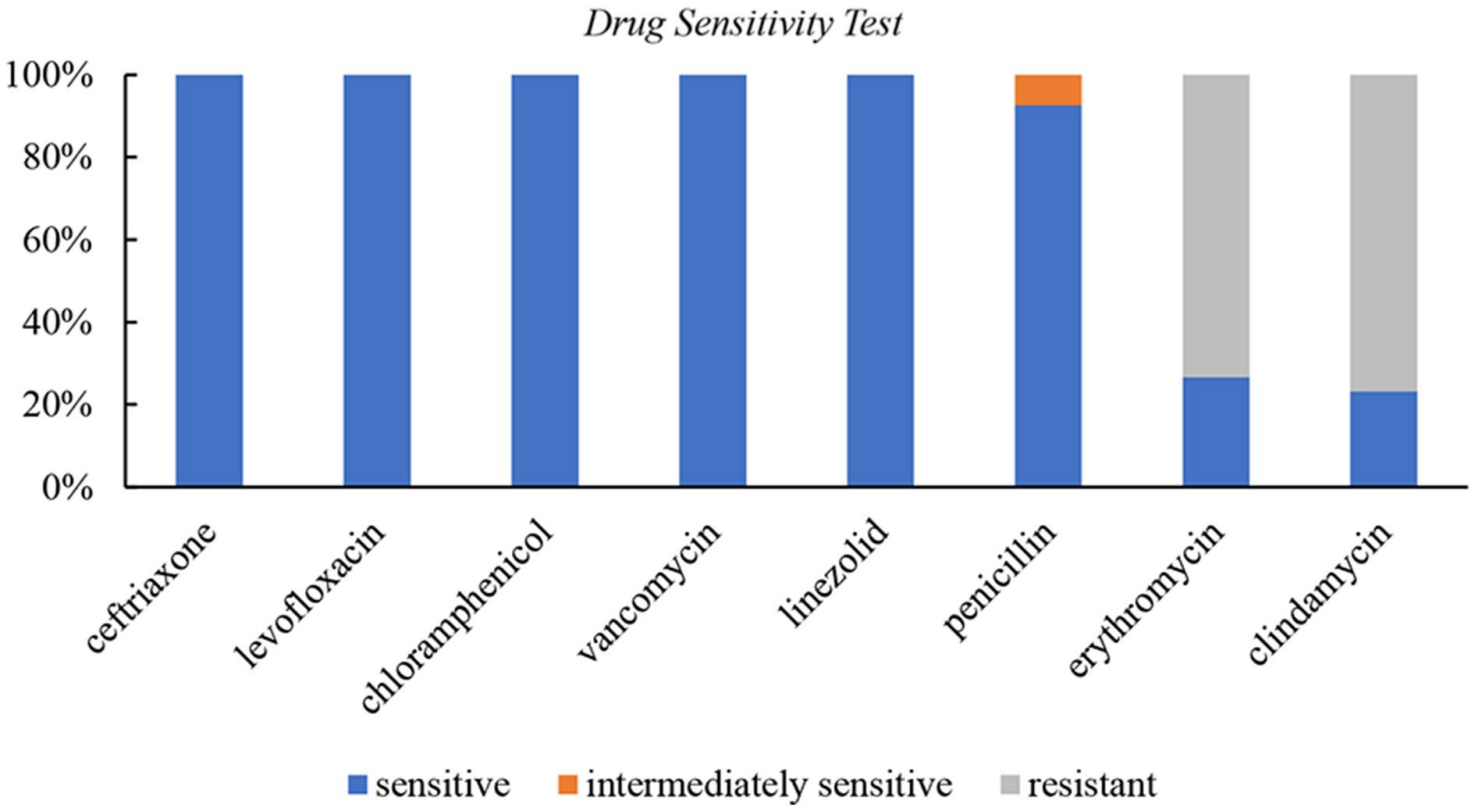
Drug sensitivity test for *S. intermedius*.

A total of 30 cases underwent susceptibility testing (Figure 2). The results showed that *S. intermedius* isolates were 100% sensitive to ceftriaxone, levofloxacin, chloramphenicol, vancomycin, and linezolid. Among the tested isolates, 8 (26.7%) were sensitive and 22 (73.3%) were resistant to erythromycin, while 7 (23.3%) were sensitive and 23 (76.7%) were resistant to clindamycin. Three cases did not undergo a penicillin susceptibility test. Among the remaining 27 cases, 25 (92.6%) were sensitive to penicillin, and 2 (7.4%) showed moderate sensitivity. *Streptococcus pneumoniae* (ATCC49619) was used as the quality control strain.

### Site of *Streptococcus intermedius* infection

Among the 40 patients, 15 had abdominal infections (14 with purulent appendicitis and 1 with an interintestinal abscess), 16 had soft tissue infections (including 9 cases affecting soft neck tissue, 2 cases affecting eye soft tissue, 1 case affecting maxillofacial area, 1 case affecting vulva, 1 case affecting perianal region, 1 case affecting sacrococcygeal region, and 1 case affecting chest wall), 5 had intracranial infections (all of which were brain abscesses), and 4 had invasive lung infections.

**Figure 3:**
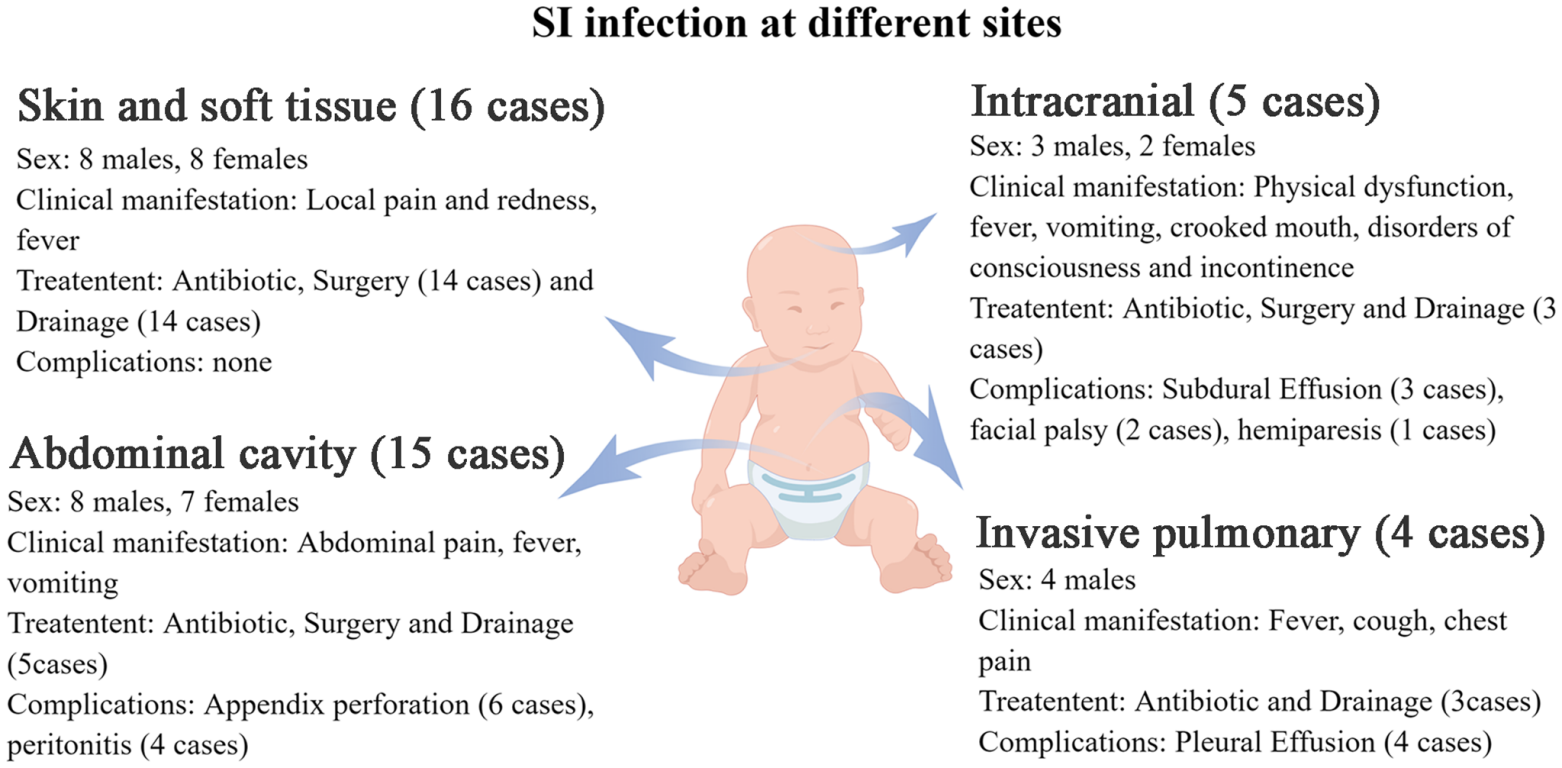
The clinical manifestations of *S. intermedius* infection at different sites in children (by Figdraw).

Additional clinical manifestations were analyzed for patients with abdominal cavity, skin and soft tissue, intracranial and lung infections (Figure 3). Abdominal infections primarily presented with abdominal pain (100%), fever (53.3%), and vomiting (33.3%). Skin and soft tissue infections mainly exhibited local pain (100%), redness (100%), swelling (100%), and fever (50%). Intracranial infections were characterized by limb movement disorders (80%), fever (60%), vomiting (60%), crooked corners of the mouth (40%), impaired consciousness (40%), and urinary and urine incontinence (20%). Invasive lung infections were characterized by fever (75%), cough (75%), and chest pain (50%).

### Antibiotics used for treatment

The antibiotics administered to children with intra-abdominal infections were mainly included third-generation cephalosporins (*n* = 13/15) and metronidazole (*n* = 13/15). Children with skin and soft tissue infections were primarily treated with third-generation cephalosporins alone (*n* = 8/16) or in combination with metronidazole (*n* = 4/16), vancomycin (*n* = 3/16), or linezolid (*n* = 1/16). Third-generation cephalosporins (*n* = 3/5), and meropenem (*n* = 4/5) were the main antibiotics used for children with intracranial infections, often combined with vancomycin (*n* = 3/5) or linezolid (*n* = 4/5). Third-generation cephalosporins (*n* = 3/4) combined with linezolid (*n* = 4/4) were the primary antibiotics used in children with invasive lung infections. In addition to antibiotic treatment, most children underwent surgery and/or drainage therapy.

### Complications encountered during treatment

The overall prognosis of the children was good, with most of them being cured and discharged from the hospital. Some patients were discharged after symptom improvement and continued oral medication. Complications encountered during treatment were showed in Figure 3. All complications were either cured or improved after treatment.

### Follow-up

Among children with intraperitoneal infection, 1 case developed a fever 1 month later but was cured after receiving appropriate anti-infection treatment. The remaining children did not experience fever, hospitalization, or the need for additional surgery. Two children developed skin and soft tissue infections, including recurrent perianal infection (*n* = 1) and recurrent neck abscess (*n* = 1). Three children underwent fistulectomy due to pyriform sinus fistula (*n* = 1) and branchial fistula (*n* = 2). For patients with intracranial infection, although head magnetic resonance imaging showed local focal lesions (*n* = 3/5), gliosis (*n* = 2/5), and slightly thickened meninges (*n* = 2/5), their speech and motor development were similar to their peers. These five patients were assessed with the “Infant-Junior Middle School Student’s Ability of Social Life Scale,” which includes six behavioral domains: self-help, locomotion, oceupation, communication, socialization, and self-direction. The total scores of all subjects were above 8 (9 scores in 1 child, 10 scores in 3 children, and 11 scores in 1 child), indicating that all subjects had social skills comparable to children with same age. For children with invasive lung infections, CT indicated significant improvement in inflammation and abscesses, and pleural effusion was absorbed after 2 months.

### Other clinical data

**Figure 4:**
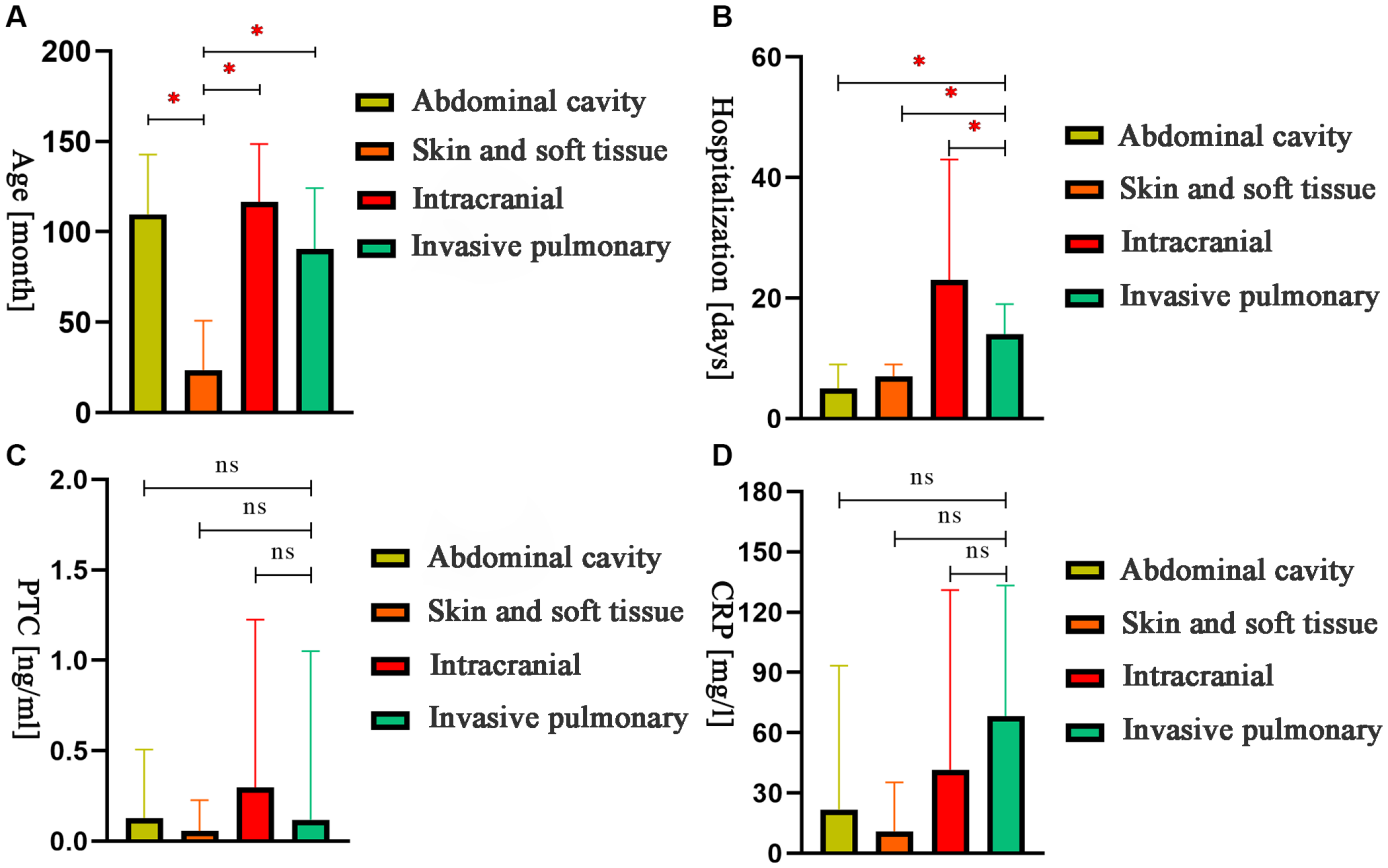
The comparison of age, hospitalization days, procalcitonin, and C-reactive protein values for children with different sites of infection. **(A)** The comparison of month-olds of different sites. **(B)** The comparison of hospital stays at different sites. **(C)** The comparison of PCT of different sites. **(D)** The comparison of CRP of different sites. *p < 0.05; ns: nonsignificant.

A comparison of age, hospitalization days, PCT levels, and CRP values was performed. The results showed that children with skin and soft tissue infections were significantly younger than those with infections in other areas (Figure 4A). Hospital stays for children with intracranial infections were significantly longer than those with invasive lung infection. Furthermore, hospital stays for children with abdominal and skin and soft tissue infections were significantly shorter than those with invasive lung infection (Figure 4B). There were no significant differences in PCT and CRP values (Figures 4C,D).

### Invasive lung infections

The detials of the 4 cases with invasive lung infections was shown in Table 2. None of them had previously reported risk factors. Chest CT scans revealed lung inflammation, combined with abscesses, nodules, or enveloping pleural effusions. It was worth noting that 2 cases yielded positive pleural high-throughput sequencing but negative bacterial cultures. The antibiotics administered mainly included ceftriaxone sodium, cefoperazone sulbactam sodium, amoxicillin clavulanate potassium, or linezolid. All cases experienced pleural effusions and required bronchoalveolar lavage, thoracentesis, or closed chest drainage in addition to anti-infective therapy.

**Table 2:** Four cases of pulmonary abscess in children with *S. intermedius* infection in our hospital.

Age (years)	Sex	Clinical manifestation	Chest computed tomography	Microbiologic Diagnosis	Antibiotic	Other treatment
10.4	Male	Chest pain	Left lower lobe nodule with adjacent pleural thickening, possibly inflammatory lesion	Pus culture and pleural fluid high throughput	Amoxicillin/Clavulanate Potassium for 7 days and linezolid for 9 days	Pleural puncture and tracheoscopy lavage
5.3	Male	Fever, cough	Abscess in the upper lobe of the right lung with peripheral pneumonitis and pleural effusion on the right	Lavage fluid culture and lavage high throughput	Ceftriaxone sodium for 10 days, linezolid for 10 days, Amoxicillin/Clavulanate Potassium for 2 days	Tracheoscopy lavage
9.6	Male	Fever, cough, chest pain	Right lower lobe inflammation with right encapsulated pleural effusion	Pleural fluid high throughput	Cefoperazone sulbactam sodium for 10 days and linezolid for 7 days	Pleural puncture
1.8	Male	Fever, cough	Pneumonia with right encapsulated pleural effusion with right middle lung atelectasis and abscess	Pleural fluid high throughput	Ceftriaxone sodium for 6 days, linezolid for 15 days	Pleural puncture and drainage

## Discussion

*Streptococcus intermedius* is one of the most common pathogens associated with brain abscess, liver abscess, and empyema. While *S. intermedius* infection is typically found in individuals aged 50–80 (Issa et al., 2020), it can also affect children. In this retrospective study, we analyzed the clinical data of pediatric patients infected with *S. intermedius* to examine the clinical features of this infection in children. In this study, we found that *S. intermedius* infection primarily affects the abdominal cavity, skin and soft tissue, intracranial, and invasive pulmonary. Unlike adults, children with *S. intermedius* infection had favorable outcomes after effective antibiotic treatment and surgical treatment or puncture drainage of lesions. Through detailed analysis of four cases of invasive lung infections, we found that two of them were negative for bacterial culture but positive for high-throughput sequencing. The clinical manifestations, treatment, and efficacy also corresponded to those of *S. intermedius* infection. This suggest that for children with pulmonary abscesses and pleural effusion, even if the bacterial culture is negative, it is necessary to be alert to *S. intermedius* infection and recommend to perform high-throughput sequencing examination.

*S. intermedius* infection can cause abscesses in multiple organs, with cerebral abscess (Mishra and Fournier, 2013; Issa et al., 2020; Sinha et al., 2021) and liver abscess (Livingston and Perez-Colon, 2014) being the most common. Other manifestations include lung abscess (Noguchi et al., 2014; Trabue et al., 2014), necrotizing pneumonia, intraperitoneal infections, bacteremia and infectious endocarditis, purulent pericarditis (Presnell et al., 2014), osteoarticular infections, spinal infections, and rarely, Lemierre syndrome (intrajugular venous septic thrombophlebitis; Tran et al., 2008). Among the 48 reported cases of *S. intermedius* infection in children (Faden, 2016), abscesses affecting the head, neck, and upper chest were the most common (52.0%), followed by intra-abdominal infection (14.5%) and limb infection (14.5%). However, among the 463 positive SAG cases reported by Jiang et al. (2020), *S. intermedius* accounted for 7.77%, among which only 11.1% were children with *S. intermedius* infection, and the abdominal cavity was the most commonly affected site, with the appendix being the most susceptible site, followed by the ear, nose and throat, and neck. The present study found that the appendix in the abdominal cavity was the most susceptible to *S. intermedius* infection, followed by soft skin tissue (mainly neck), intracranial, and lung invasiveness, consistent with Jiang et al.’s results (Jiang et al., 2020). SAG bacteremia has received increasing attention in recent years, with the incidence rate increasing from 0.93/100,000 (population-based study from 1989 to 2000; Weightman et al., 2004) to 3.7/100,000 (epidemiological reports from 2010 to 2017; Laupland et al., 2018), and the mortality rates of SAG-related bacteremia patients ranging between 10 to 16% (Issa et al., 2020). A single-center clinical study suggested that elderly patients (>63 years) and solid tumors were high-risk factors for SAG bacteremia (Isa et al., 2022). Compared to other SAG species, *S. intermedius* has a higher tendency to form abscesses and deep tissue infections and is less likely to cause bacteremia. In our study, we identified one case of positive blood culture for *S. intermedius*. However, since the child’s follow-up blood culture was negative and did not exhibit any symptoms of *S. intermedius* infection (such as fever or abscess), we excluded this case from the analysis.

The clinical manifestations of *S. intermedius* infection varied depending on the affected body part. In cases of intracranial infection, limb movement disorders, fever, and vomiting were the dominant manifestations. Infections in other body parts were primarily characterized by local pain and fever, consistent with the clinical characteristics of abscess formation. A comparison of infections in different body parts revealed that children with skin and soft tissue infections were significantly younger than those in other parts. Children with intracranial infection had a longer hospital stay, likely due to the presence of the blood–brain barrier (Zhao et al., 2015), which necessitates prolonged antibiotic courses. On the other hand, children with abdominal and neck soft tissue infections could undergo timely and effective surgical and drainage treatments, leading to significantly shorter hospital stays than those with lung infections.

The choice, duration, and route of antibiotic treatment for *S. intermedius* infections are based on limited evidence. *S. intermedius* is usually sensitive to β-lactams (Cobo et al., 2018), and some strains exhibit mild sensitivity or resistance to penicillin. Vancomycin is an appropriate alternative treatment in cases where β-lactams cause allergic or resistant reactions while the resistance to macrolides appears to be increasing. The UK Society of Antimicrobial Chemotherapy guidelines (Darlow et al., 2020) recommend using cefotaxime or ceftriaxone in combination with metronidazole for *S. intermedius* brain abscess; however, these recommendations may not be appropriate for pediatric patients. Summarizing the previous reports of 21 cases of intracranial abscess caused by *S. intermedius* infection in children, it was found that only 4 of them had co-infection with anaerobic bacteria, and only 6 of them were treated with metronidazole (Issa et al., 2020). The duration of antibiotic treatment is typically 6 to 8 weeks, depending on the causative organism and its resistance pattern (Brouwer et al., 2014). Furuichi and Horikoshi (2018) reported that *S. intermedius* showed 100% sensitivity to penicillin, ampicillin, cefotaxime, erythromycin, clindamycin, levofloxacin, and vancomycin in pediatric cases. In our study, *S. intermedius* showed sensitivity to third-generation cephalosporins, vancomycin, and linezolid, as well as good overall sensitivity to penicillin antibiotics, but resistance to macrolides. These findings provide a basis for empirical antibiotic treatment choices in children with *S. intermedius* infection.

In addition to antibiotic therapy, abscess drainage and surgical removal are crucial in treating *S. intermedius* infections. Surgery should be considered for cases involving multiple sites, fungal infections, traumatic abscesses, or when there is no response to treatment within 1 week (Al Moussawi et al., 2018). Complete surgical drainage is recommended for abscesses larger than 2.5 cm. Issa et al. (2020) summarized 101 cases of *S. intermedius*-induced brain abscesses, with 92.2% of patients recovering after treatment. Cobo et al. (2018) reported 15 cases of pleural lung infections caused by *S. intermedius*, of which 10 were cured with medical and surgical treatment, while 2 died from the infection. In our study, among the 34 cases treated with effective antibiotics and concurrent surgery or puncture drainage of lesions (82.9%), none died during hospitalization. Although there were some complications, such as appendix perforation and subdural effusion, the relevant symptoms significantly improved after treatment, indicating that effective anti-infective therapy and early surgical treatment/drainage therapy led to favorable outcomes in most children.

Pleural effusion or empyema caused by *S. intermedius* infection is not uncommon, particularly in adults with risk factors such as smoking, alcohol abuse, periodontal disease, chronic obstructive pulmonary disease and diabetes mellitus (Hannoodi et al., 2016; Cobo et al., 2018). Previous studies have described imaging findings in patients with *S. intermedius* infection, including pleural effusions, cavities, nodules, empyema, and lung abscesses (Tasleem et al., 2021). However, reports of pleural effusion or empyema caused by *S. intermedius* infection in children are relatively rare. To our knowledge, only one case (Nakagawa et al., 2022) has hitherto been reported. Our study summarized four cases of pleural effusion/empyema caused by *S. intermedius* infection in children. High-throughput sequencing was more sensitive in detecting *S. intermedius* in children with lung invasive infections than bacterial culture (50% positive rate). High-throughput sequencing is a comprehensive method based on the sequence identification of pathogenic microorganisms (Graff et al., 2021; Vestergaard et al., 2021), and previous studies have demonstrated its advantages in detecting *S. intermedius* infections in brain abscesses (Hu et al., 2019; Stebner et al., 2021) and pleural empyema (Dyrhovden et al., 2019). It serves as an effective complement to traditional methods such as bacterial culture. Therefore, even if bacterial culture results are negative, *S. intermedius* infection should be considered in children with concurrent lung abscess and pleural effusion, especially in boys. Performing high-throughput nucleotide sequencing can help reduce misdiagnosis rates.

The present study has some limitations. This retrospective study was limited by its single-center design and relatively small sample size, which may limit the generalizability of the results to other populations.

In summary, *S. intermedius* infection primarily affects the abdominal cavity, skin and soft tissue, intracranial region, and lungs. The formation of abscesses is a common manifestation of *S. intermedius* infection, making it necessary to consider this pathogen when pus specimens or abscesses are detected. *S. intermedius* showed sensitivity to third-generation cephalosporins, vancomycin, linezolid, and penicillin antibiotics, while resistance to macrolides was observed. Most children had favorable outcomes with aggressive anti-infective therapy combined with surgical treatment or puncture drainage of lesions. Pleural effusions or empyema caused by *S. intermedius* infection are not uncommon in children, and even in cases where cultures are negative, *S. intermedius* infection should be considered in children with lung abscesses and pleural effusions, especially boys. High-throughput nucleotide sequencing can be a valuable tool to reduce misdiagnosis rates.

## Data availability statement

The original contributions presented in the study are included in the article/supplementary material, further inquiries can be directed to the corresponding author.

## Ethics statement

The studies involving human participants were reviewed and approved by the Medical Ethics Committee of children’s hospital affiliated with Zhejiang University School of Medicine. Written informed consent from the participants’ legal guardian/next of kin was not required to participate in this study in accordance with the national legislation and the institutional requirements.

## Author contributions

ZX and LG designed the project, collected data, drafted the initial manuscript, and revised the manuscript. DX and DY collected data, performed the statistical analysis, and revised the manuscript. ZC and YW conceptualized and designed the study and reviewed and revised the manuscript. All authors contributed to the article and approved the submitted version.

## Funding

This research was supported by the National Key R&D Program of China (grant number: 2019YFE0126200) and the National Natural Science Foundation of China (grant number: 62076218).

## Conflict of interest

The authors declare that the research was conducted in the absence of any commercial or financial relationships that could be construed as a potential conflict of interest.

## Publisher’s note

All claims expressed in this article are solely those of the authors and do not necessarily represent those of their affiliated organizations, or those of the publisher, the editors and the reviewers. Any product that may be evaluated in this article, or claim that may be made by its manufacturer, is not guaranteed or endorsed by the publisher.
